# Supplementary material for: Synergistic Role of Amino Acids in Enhancing mTOR Activation Through Lysosome Positioning
Source: bioRxiv. 2024 Oct 13:2024.10.12.618047. Preprint. [Version 1] doi: 10.1101/2024.10.12.618047 (PMC11482915; doi:10.1101/2024.10.12.618047)
Supplement: Supplement 1 [file media-1.pdf]

SUPPLEMENTAL INFORMATION

Supplemental figure legends

Fig. S1. Amino acids regulate endosome and lysosome positioning

A. Monkey kidney fibroblast-like cells COS-7 and human osteosarcoma U2OS cells were starved in amino acid-free DMEM media (with dialyzed serum and glucose) for 1 h, subjected to immunofluorescence with the indicated antibodies, and imaged by confocal microscopy. Yellow doted lines were manually added to indicate cell boundaries. **B.** HeLa cells were incubated in complete media, amino acid- and serum-free DMEM (Starvation) for 1.5 h, or 2 mM phenylalanine in amino acid- and serum-free media for 30 minutes following 1 h starvation. The indicated antibodies were applied in immunofluorescence, and images were collected by confocal microscopy. Scale bars, 5 μm.

Fig. S2. The effect of amino acids on lysosome retrograde transport

A. Wildtype (WT) and Rab7-KO HeLa cells were fixed and co-stained with filipin (for free cholesterol) and the indicated antibodies in detergent-free saline buffer. Confocal microscopy was performed for imaging. **B**. HeLa cells were pretreated with DMSO or 0.5 μM TRPML1 agonist ML-SA5 for 1 h, starved in amino acid- and serum-free DMEM with DMSO or ML-SA5 for 1 h, and refed with 2 mM individual indicated amino acids accompanied with DMSO or ML-SA5 for 30 min. Cells were subjected to immunofluorescence and analyzed for lysosome positioning as described in Methods. p values were determined using *Student's t* test (vs. the corresponding point in the control group). ns, not significant. Scale bars, 5 μm.

Fig. S3 Synergic effect of amino acids on mTOR activation

A. HeLa cells were starved in amino acid- and serum-free DMEM for 1 h and refed with 2 mM individual or combined indicated amino acids for 30 min. Cells were subjected to immunoblotting with the indicated antibodies. The ratio of p-S6K to total S6K for each treatment was normalized to the values in the starvation group. Individual amino acid treatments were summed (labeled as "+") and compared to the corresponding values from the combination of the two amino acids (labeled as "&"). Paired values from three independent experiments were shown with lines connecting the data points. B. Wildtype (WT) and the indicated KO cells (SLC: SLC38A9. Kin: kinesin1/3) were treated

as in A with combined amino acids. Paired values from two independent experiments were shown with lines connecting the data points. **C.** HeLa cells were starved in amino acid- and serum-free DMEM for 1 h and refed with 2 mM individual or combined indicated amino acids for 30 min. Cells were subjected to immunofluorescence and analysis of lysosome positioning as described in Methods. *p* values were determined using one-way ANOVA (vs. Starvation group) or *Student's t* test (indicated with lines). **, $p<0.01$ ***, $p<0.001$ ****, $p<0.0001$. ns, not significant.

Supplemental tables

Table 1. Amino acid concentrations in DMEM and adults' blood

Amino Acid	Sigma Cat#	Stock concentration (mM)	Concentration in DMEM (mM)	Concentration in blood (mM)
L-Glutamine	A2916801 (ThermoFisher)	200	4	0.4-0.8
Glycine	G7126	200	0.4	0.12-0.55
L-Arginine	11009	200	0.4	0.02-0.14
L-Cystine dihydrochloride	C6727-25G	(Working concentration)	0.2	0.0008-0.028
L-Histidine	H8000	200	0.2	0.057-0.114
L-Isoleucine	W527602	200	0.8	0.038-0.13
L-Leucine	61819	100	0.8	0.074-0.196
L-Lysine hydrochloride	L8662	200	0.8	0.12-0.318
L-Methionine	64319	200	0.2	0.014-0.148
L-Phenylalanine	P2126	100	0.4	0.035-0.085
L-Serine	S4500	200	0.4	0.06-0.172
L-Threonine	T8625	200	0.8	0.073-0.216
L-Tryptophan	T3300	50	0.08	0.031-0.083
L-Tyrosine disodium salt dihydrate	RES3156T-A7	200	0.4	0.03-0.12
L-Valine	94619	200	0.8	0.146-0.37

Table. S2 Antibodies used in this study

Antibody	Source	Product Number	Dilution	Applications
Actin	BD Bioscience	612657	1:10,000	WB
Calnexin	Cell Signaling Technologies	2679s	1:8,000	WB
COXIV	Cell Signaling Technologies	11967S	1:200	IF
EEA1	Cell Signaling Technologies	3288	1:100	IF
JIP4	Cell Signaling Technologies	5519s	1:100, 1:1,000	IF, WB
LAMP1	Cell Signaling Technologies	9091s	1:1,000	IF
LAMP2	Santa Cruz	SC-18822	1:500	IF
LAMTOR4	Cell Signaling Technologies	13140S	1:200	IF
LC3	MBL	PM036	1:200	IF
PLIN2	Santa Cruz	sc-377429	1:100	IF
Rab5	Cell Signaling Technologies	3547	1:100	IF
Rab7a	Cell Signaling Technologies	9367	1:100, 1:1000	IF, WB
p-S6K	Cell Signaling Technologies	9234S	1:1,000	WB
S6K	Cell Signaling Technologies	34475S	1:1,000	WB
TGN46	BioRad	AHP500GT	1:1000	IF

Fig. S1

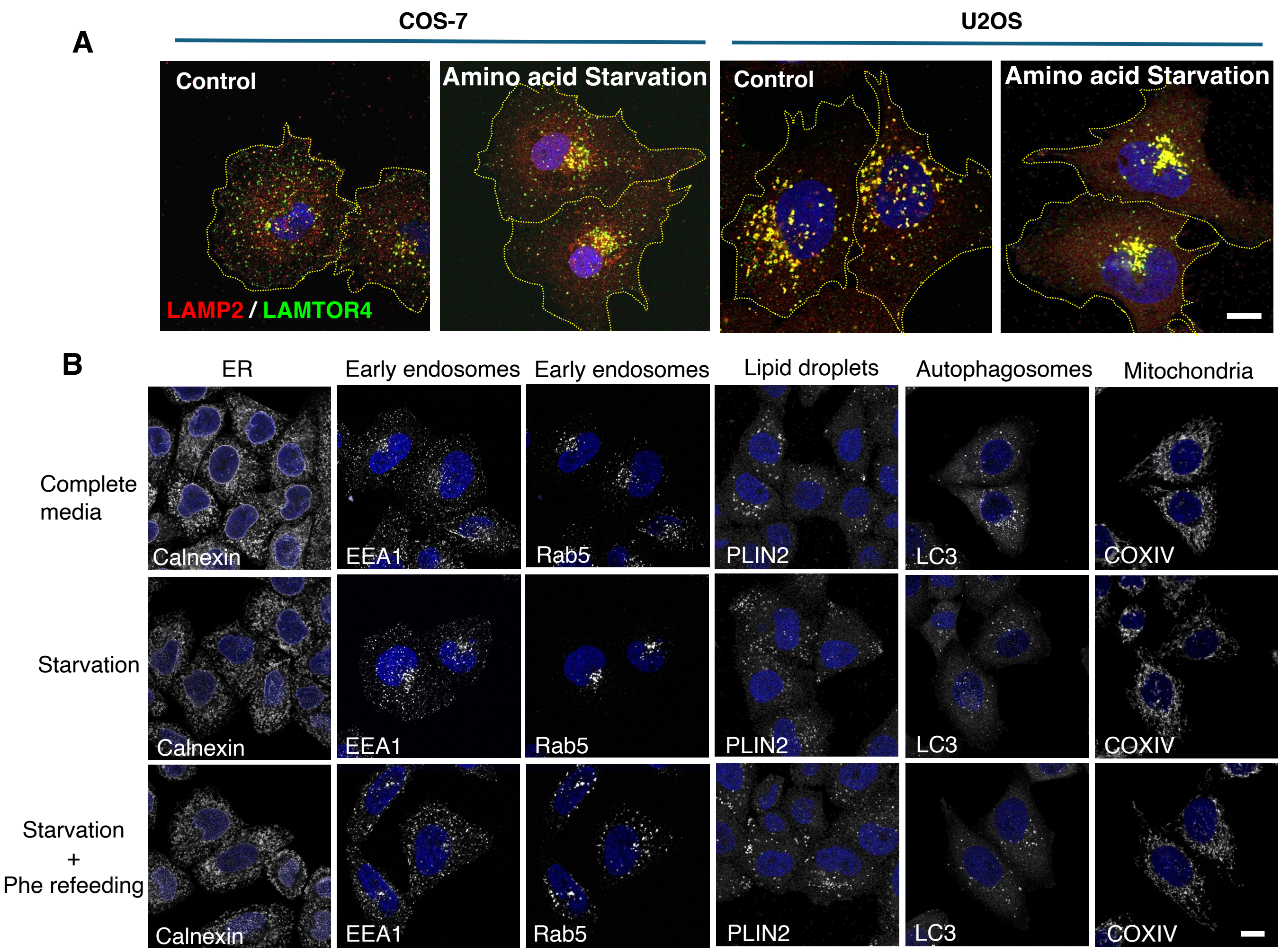
A
COS-7
U2OS
Control
Amino acid Starvation
Control
Amino acid Starvation
LAMP2 / LAMTOR4
B
ER
Early endosomes
Early endosomes
Lipid droplets
Autophagosomes
Mitochondria
Complete media
Starvation
Starvation + Phe refeeding
Calnexin
EEA1
Rab5
PLIN2
LC3
COXIV

Fig. S2

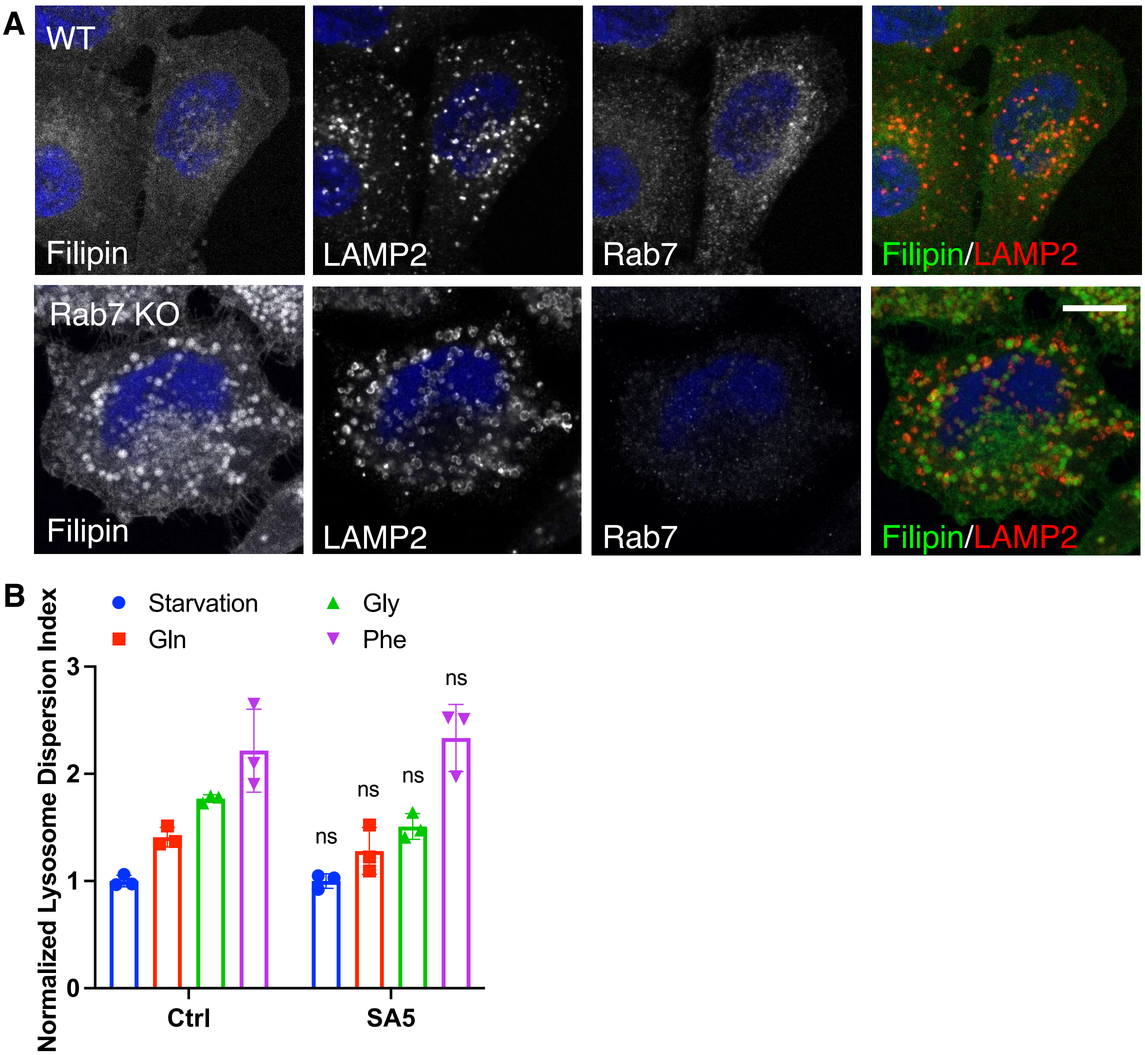

A
WT
Filipin
LAMP2
Rab7
Filipin/LAMP2
Rab7 KO
Filipin
LAMP2
Rab7
Filipin/LAMP2
B
Starvation
Gln
Gly
Phe
Normalized Lysosome Dispersion Index
0
1
2
3
ns
ns
ns
ns
Ctrl
SA5

Fig. S3

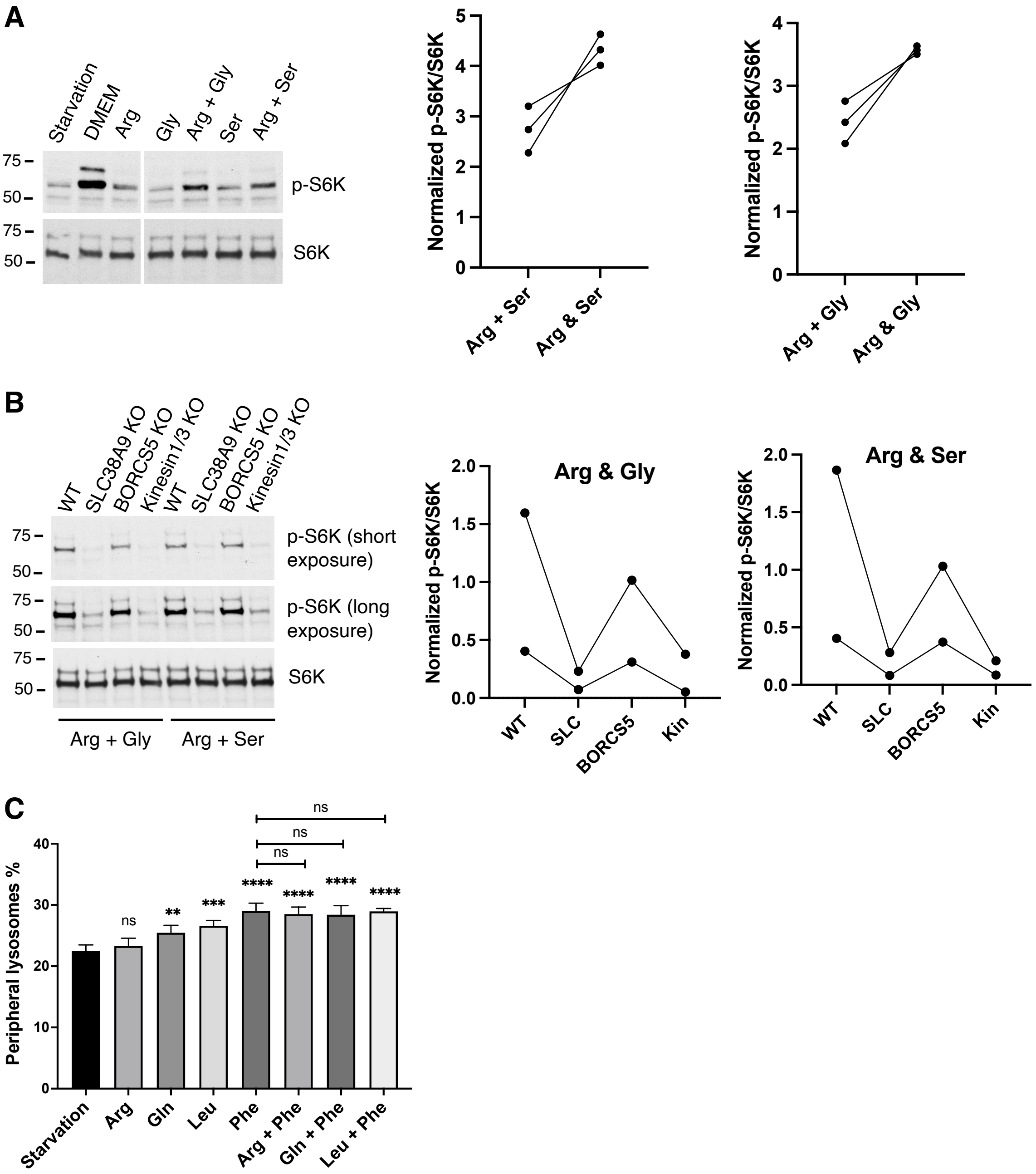

A
Starvation
DMEM
Arg
Gly
Arg + Gly
Ser
Arg + Ser
75
50
75
50
p-S6K
S6K
Normalized p-S6K/S6K
Arg + Ser
Arg & Ser
Arg + Gly
Arg & Gly
B
WT
SLC38A9 KO
BORCS5 KO
Kinesin1/3 KO
p-S6K (short exposure)
p-S6K (long exposure)
S6K
Arg + Gly
Arg + Ser
Arg & Gly
Arg & Ser
SLC
BORCS5
Kin
C
Peripheral lysosomes %
ns
**
***
****
Starvation
Arg
Gln
Leu
Phe
Arg + Phe
Gln + Phe
Leu + Phe
